# Predator versus Prey: Locust Looming-Detector Neuron and Behavioural Responses to Stimuli Representing Attacking Bird Predators

**DOI:** 10.1371/journal.pone.0050146

**Published:** 2012-11-27

**Authors:** Roger D. Santer, F. Claire Rind, Peter J. Simmons

**Affiliations:** 1 Institute of Biological, Environmental, and Rural Sciences, Aberystwyth University, Aberystwyth, United Kingdom; 2 Department of Life Sciences, University of Limerick, Limerick, Ireland; 3 Institute of Neuroscience, Newcastle University, Newcastle upon Tyne, United Kingdom; University of Sussex, United Kingdom

## Abstract

Many arthropods possess escape-triggering neural mechanisms that help them evade predators. These mechanisms are important neuroethological models, but they are rarely investigated using predator-like stimuli because there is often insufficient information on real predator attacks. Locusts possess uniquely identifiable visual neurons (the descending contralateral movement detectors, DCMDs) that are well-studied looming motion detectors. The DCMDs trigger ‘glides’ in flying locusts, which are hypothesised to be appropriate last-ditch responses to the looms of avian predators. To date it has not been possible to study glides in response to stimuli simulating bird attacks because such attacks have not been characterised. We analyse video of wild black kites attacking flying locusts, and estimate kite attack speeds of 10.8±1.4 m/s. We estimate that the loom of a kite’s thorax towards a locust at these speeds should be characterised by a relatively low ratio of half size to speed (*l/|v|*) in the range 4–17 ms. Peak DCMD spike rate and gliding response occurrence are known to increase as *l/|v|* decreases for simple looming shapes. Using simulated looming discs, we investigate these trends and show that both DCMD and behavioural responses are strong to stimuli with kite-like *l/|v|* ratios. Adding wings to looming discs to produce a more realistic stimulus shape did not disrupt the overall relationships of DCMD and gliding occurrence to stimulus *l/|v|*. However, adding wings to looming discs did slightly reduce high frequency DCMD spike rates in the final stages of object approach, and slightly delay glide initiation. Looming discs with or without wings triggered glides closer to the time of collision as *l/|v|* declined, and relatively infrequently before collision at very low *l/|v|*. However, the performance of this system is in line with expectations for a last-ditch escape response.

## Introduction

The roles of identifiable neurons in triggering behaviour are particularly well understood in fast, reliable escape-triggering mechanisms such as those of several arthropods [1]. Such mechanisms presumably evolved as a result of selection pressures exerted by natural predators. However, the characteristics of natural predator attacks, and the stimuli that they would provide to their prey, have rarely been described [2], [3], [4], [5]. This means that it is often not possible to investigate escape-triggering mechanisms within the context of escape from a real predator. Where escape-triggering mechanisms have been studied during real or simulated predator attacks, these mechanisms confer a variable probability of successful escape, around 50% or less in many investigations [6], [7], [8], perhaps reflecting the finely balanced arms-race between predator and prey. Here we attempt to characterise the attacks of a natural predator of flying locusts. We then use simulated stimuli representing these attacks to investigate emergency behavioural responses triggered by a locust’s descending contralateral movement detector (DCMD) neuron, one of the most frequently studied identifiable visual neurons of invertebrates.

When a predator approaches, it can be perceived as a looming visual stimulus: its image expands over the eye of the viewer with a rate that increases as the time of collision nears. Looming-sensitive visual neurons have been found in a huge range of taxa, both vertebrate and invertebrate [9], [10], [11], [12], [13], [14], [15], [16], but best understood among these is the single bilateral pair of DCMD neurons of acridid grasshoppers (including locusts) [17], [18]. Each DCMD has its cell body in the protocerebrum of the brain, and its axon descends to the thoracic ganglia where it excites neurons controlling leg and wing movements [19], [20], [21]. Input to each DCMD is from a uniquely identifiable lobula giant movement detector (LGMD) neuron [22], which collects visual input from most of the visual field of one compound eye in the lobula of the optic lobe [23]. It is at the LGMD that selectivity for looming arises (e.g. [24], [25]), but it is more convenient to record from the axon of the DCMD, in which spikes follow those in the LGMD one-for-one [26]. The DCMD responds most strongly to objects approaching on a direct collision course, and much less strongly to objects moving along non-collision trajectories [27]. The DCMD response tracks object approach, producing a train of spikes that increases in frequency as a looming object expands over the locust’s eye [17], [28]. For a simple looming disc or square, the profile of expansion depends on the ratio of the object’s half size to its approach speed (*l/|v|*), and peak DCMD spike rate increases as *l/|v|* decreases [29], [30]. The time of the peak DCMD response depends linearly on stimulus *l/|v|*; it occurs earlier before collision as *l/|v|* increases, regardless of the object’s actual size or speed [29], although DCMD peak response timing is affected by arousal [31].

Startle responses to looming stimuli are common across animal taxa, and several recent studies have explored the link between identified looming-sensitive neurons and emergency behavioural responses using the locust DCMD neuron (for reviews, [32], [33]). A number of different features of the DCMD spike train are involved in the production of escape jumps in locusts on the ground [34], [35], [36]. In flying locusts, high-frequency bursts of DCMD spikes cause a gliding response [37], [38], and this is the behaviour of concern in the current paper. When a tethered flying locust experiences a looming stimulus, it attempts to steer away from the developing threat as its initial escape response [37], [39], [40], [41]. If the stimulus continues to approach, the locust performs a last-ditch ‘glide’: it reliably ceases to beat its wings and raises them into a stereotyped swept-back posture, only a few milliseconds before collision [37], [39]. Tonic contraction of a forewing elevator muscle, M84, is a signature of glide occurrence. DCMD spikes directly excite the motor neuron of this muscle, and when DCMD spikes occur at >150 Hz in restrained locusts, excitation of the motor neuron is sufficient to cause it to spike and for the muscle to contract [38]. During tethered flight, >150 Hz DCMD spikes appear to be effective in eliciting glides only when they coincide with wing elevation, most likely due to gating of the behavioural response by ongoing rhythmic modulation of flight motor neuron membrane potential [38]. As such, gliding responses are variable in occurrence and timing, but are more frequently observed in response to faster looming stimuli (with lower *l/|v|*), which elicit higher DCMD peak spike rates [37]. However, when the connective contralateral to the eye viewing a looming stimulus is severed so that DCMD spikes cannot reach the meso- or metathoracic ganglia, glides do still occur occasionally [38]. Therefore, it may be that looming-sensitive neurons descending in the ipsilateral connective, such as the descending ipsilateral movement detector (DIMD) [20], also contribute to glide triggering. Glides triggered by stimuli looming from the side are distinct from landing attempts because the locust’s legs remain tucked into its body and flying often resumes quickly, and these may be attempts to change course and evade capture by a predator [37]. When an object looms from the front, glides are accompanied by foreleg extension and it has been proposed that this might help absorb some of the force from the imminent impact, or allow landing [39].

One context in which the DCMD and the glides it triggers may function is in the detection and evasion of bird predators [37], [42], [43]. Swarming desert locusts are preyed upon by predatory birds of a variety of species, both during flight and when on the ground [44], [45], [46], [47]. Although birds can take large numbers of locusts, their overall impact on locust numbers depends on population density [44]. Bird predators also have a variable impact on rangeland grasshopper numbers in the USA [48], [49]. Black kites, *Milvus migrans*, are one predatory bird species regularly reported attacking swarming, flying locusts in Africa and Australia [46], [47], [50]. These birds may be the commonest raptor in the World [51], overlapping the natural ranges of the two locust species commonly used in laboratory studies (*Schistocerca gregaria* and *Locusta migratoria*). During locust outbreaks, black kites form large foraging groups that may be >100 individuals strong [47]. Although black kites are generalists, when the opportunity occurs they prey heavily on locusts: during a desert locust outbreak in the Sahel 100% of sampled kite pellets contained locust remains, whereas 4% of sampled pellets did before the outbreak [47].

The attack behaviour of bird predators of flying locusts has not been quantified, and thus DCMD performance in triggering gliding responses to stimuli that resemble these attacks could not be investigated. Complex bird-like outlines have been used to stimulate the DCMD [52], but in these experiments the focus was on understanding the process of habituation to repeated approaches, and behavioural responses were not investigated. Among the simple stimuli used in most laboratory experiments, it has been suggested that those with lower *l/|v|* ratios (smaller, faster looming objects), may be most similar to attacking bird predators [42], [43]. Although it has been shown that DCMD responses are stronger [29], [30], and gliding behaviours more frequent [37] in response to simple looming shapes with low *l/|v|*, both trends have not yet been demonstrated within the same locust species (or the same genus). More importantly, it has not been possible to interpret these trends using information on which part of the *l/|v|* range is representative of bird predator attacks, or to examine how these relationships are affected by a more realistic stimulus shape.

In this study we use video footage to characterise the attack behaviour of wild black kites on locusts flying in a swarm. We measure kite attack speeds and from them estimate a range of *l/|v|* ratios representative of looming kite thoraces. In laboratory experiments, we record DCMD and gliding responses of *L. migratoria* to computer-generated looming stimuli across a range of *l/|v|* values. Using simple looming discs, we investigate known trends in DCMD spike rate and glide occurrence with *l/|v|* for comparison with *l/|v|* ratios estimated for attacking kite thoraces. We also investigate whether glides occur before the theoretical moment of interception by the predator in response to these stimuli. By adding wings to looming discs across a variety of *l/|v|* ratios we investigate how a more bird-like stimulus shape affects the DCMD response, the probability of a glide occurring, and the probability that when a glide is performed it will be initiated before interception by the predator.

## Results

### Black Kite Behaviour

**Figure 1:**
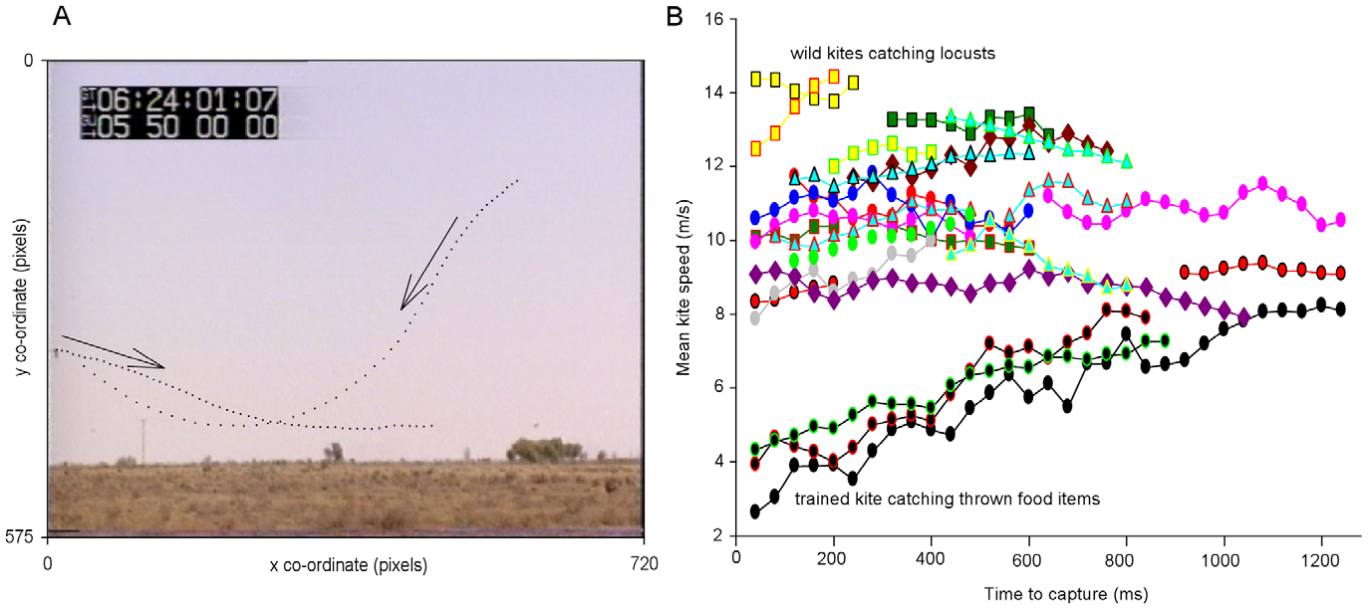
Attacks by wild black kites (*Milvus migrans*) on swarming Australian plague locusts (*Chortoicetes terminifera*). A, Typical kite trajectories during attacks on swarming, flying locusts. The head positions of two black kites are plotted in x, y coordinates at 40 ms (one frame) intervals. Kites characteristically circled above the locust swarm and made swoops into the swarm to catch locusts. Here, one kite performs a shallow swoop into low-level flapping flight (left to right); a second kite performs a steeper swoop (right to left). Arrows indicate direction of travel. The background is a wide-field image frame taken from the moment that the first kite began its attack (left of frame), and the second kite is not visible at this time. ©NHNZ Moving Images. B, Kite speeds during attacks on flying locusts, or captures of thrown prey items. Kite speeds were measured for 17 attacks on flying locusts by 10 wild kites where adequate reference features were present in frame (see methods). Common symbol shapes and fill colours indicate the same individual kite; symbol edge colours distinguish separate attacks by the same kite. Three catches of thrown food items were filmed in a captive back kite using a static camera (lower traces, circles with black fill), and for these speed could be analysed continuously. Speeds are plotted at one frame intervals, and are smoothed by averaging across the two neighbouring frames for clarity (means quoted in text were not based on smoothed data; see methods). The captive kite decelerated while catching thrown food items, but this was not clearly evident for wild kite attacks on locusts.

Black kite (*M. migrans)* attack behaviour was investigated using video footage of groups of these birds capturing flying Australian plague locusts, *Chortoicetes terminifera* (Acrididae: Oedipodinae), in Mundi Mundi, NSW, Australia. In this footage, kites characteristically circled above the swarming locusts, periodically swooping into the swarm and attempting to catch a locust using their talons. The tracks of two kites performing swooping attacks are shown in Fig. 1A (and more detailed images of kites are shown in Fig. S1, and in Fig. S2 in which blurred images of locusts are also visible). In some attacks kites glided with wings held outstretched and wrists slightly flexed, in others kites flapped their wings during pursuit or combined flapping with gliding. Bouts of attack behaviour by individual kites were very intensive, with consecutive capture attempts separated by a mean interval of 6.4±3.5 s (mean ± SD, N = 12 kites). Following an unsuccessful capture attempt, a kite characteristically began another by continuing to fly through the swarm, but following a successful capture it climbed back up to circling height where it consumed the locust on the wing, bending its head downwards and extending its legs in order to peck at the locust held in its talons. One instance was noted in which the kite clearly mishandled and dropped a locust, but still bent its head towards its talons. From unambiguous observations of feeding behaviour following a capture attempt, the proportion of capture attempts that were successful was estimated at 0.8±0.2 (N = 12). Because of the small size of locusts, it was not possible to distinguish clearly the target locust of most attacks, so their behaviour in response to attack could not be assessed. However, we did note cases in which a flying locust rapidly lost height as a kite loomed close to it (locust arrowed in later frames of Fig. S2).

From kite attacks oriented perpendicular to the camera’s direction of view, we estimated kite flight speeds for later comparison with simulated looming stimuli used in laboratory experiments. During filmed prey capture events, kite speed sampled during the final 1.25 s of attack (Fig. 1B) was 10.76±1.42 m/s (range: 8.73–13.31 m/s; N = 10 kites [17 attacks]). Since these measurements relied on video calibration by estimated mean kite length (see methods), we also filmed a trained black kite catching thrown food items using a fixed camera and exact calibration. Unfortunately, the kite’s behaviour in this scenario was qualitatively different to that observed in wild kites, and there was a clear deceleration prior to the catch that was not evident when wild kites caught flying locusts (Fig. 1B). However, kite speeds in advance of speed adjustment for capture were broadly comparable, providing some support for our measurements of wild kites from video. Due to the lack of a clear pattern of acceleration or deceleration when wild kites caught flying locusts, we made the simplifying assumption that kites attacked locusts with a relatively constant speed, and thus kite speeds would represent the closing speeds that a stationary locust would experience.

The closing speed between kite and locust would vary with kite speed, locust speed, and the angle at which their flight paths converge. The flight speeds of swarming *L. migratoria* have been measured previously [53], so we estimated a window of closing speeds for an attacking kite based on these. Since kites generally attacked along slightly downward or flat trajectories, we only considered variation in angles of flight path convergence in the horizontal plane. Closing speeds would range from 17.43 m/s for a fast kite (mean speed + SD) and a fast locust (mean speed + SD) converging head-on, to 4.09 m/s for a slow kite (mean speed – SD) converging on a fast locust (mean speed + SD) from behind. This corresponds to an *l/|v|* range of 3.9–16.6 ms for a looming kite’s thorax, as indicated on each of the following figures of experimental data.

### Locust dcmd and Behavioural Responses to Looming Discs

Laboratory experiments were performed on *Locusta migratoria*, a member of the same subfamily as *C. terminifera* (Acrididae: Oedipodinae) which also forms swarms and occurs in Australia. It can therefore be presumed that *L. migratoria* is also subject to predation by black kites.

**Figure 2:**
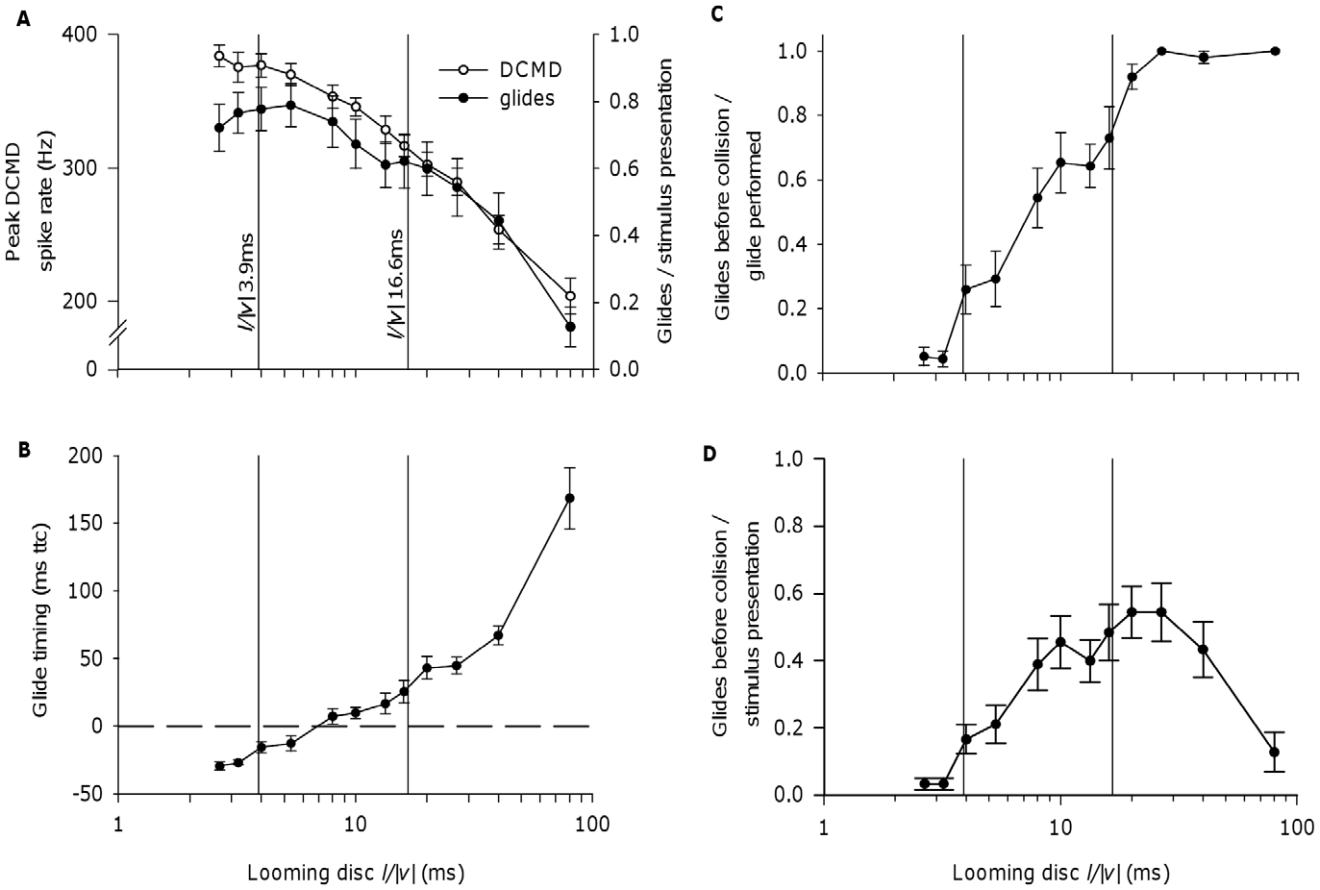
Estimated *l/|v|* for kite thoraces, and DCMD and behavioural responses to looming discs of varying *l/|v|* in *Locusta migratoria*. A, Known trends in DCMD and behavioural response properties over the range of *l/|v|* values estimated for the thoraces of looming kites (vertical lines in this and subsequent panels, see text). Both DCMD peak spike rate (open circles; as previously demonstrated for *Schistocerca* spp. [29], [30]) and gliding response occurrence (closed circles, as previously demonstrated for *L. migratoria* [37]), increased with decreasing *l/|v|* in our experiments on *L. migratoria*. Both DCMD and gliding responses were strong within the kite-like range of *l/|v|* values. B, In our experiments, the mean timing of glide initiation, relative to the predicted time of collision, declined with declining stimulus *l/|v|*, and mean glide timing was after the moment of collision (dashed line) for the looming discs with the lowest *l/|v|* ratios tested. C, Glides could still be successfully initiated before collision within the estimated range of kite-like *l/|v|* values. Plot shows the number of glides successfully initiated before the moment of collision as a proportion of all glides performed. D, The number of glides initiated before collision expressed as a proportion of stimulus presentations. Plot shows the combined effect of increasing glide occurrence with decreasing *l/|v|* (panel A, black circles), and increasing probability of glide initiation before collision with increasing *l/|v|* (panel C). DCMD response measurement in panel A: N = 10 locusts (value for each individual a mean of responses to 6 presentations of each stimulus). Glide measurements in panels A and D: N = 15 locusts (value for each individual calculated from 6 presentations of each stimulus). Glide measurements in panels B and C: Varying numbers of individuals per data point since locusts that did not glide could not be included. N at *l/|v|* 80.0 ms = 5; *l/|v|* 40.0 ms = 13; *l/|v|* 26.7, 20.0, 13.3, 10.0, 8.0, 2.7 ms = 14; *l/|v|* 16.0, 5.3, 4.0, 3.2 ms = 15. In all panels, means plotted ± SEM.

Peak DCMD spike rates [29], [30] and gliding response occurrence [37] are known to increase with decreasing stimulus *l/|v|*, but to date the two trends have been demonstrated in separate studies using locusts of different species, and the DCMD trend has not been followed through the lower limit of the *l/|v|* range we predict for kite attacks. In response to looming discs with varying *l/|v|*, peak DCMD spike rate in *L. migratoria* varied significantly with disc *l/|v|* (Fig. 2A, open circles; repeated measures ANOVA, F_1.9,17.3_ = 87.80, p = 0.001) and was greatest at low *l/|v|*, which is in agreement with previous work on *Schistocerca* spp. [29], [30]. As we showed previously in *L. migratoria* [37], but replicated here using the stimuli and display monitor that we used in electrophysiological experiments, glide occurrence also increased with declining stimulus *l/|v|* (Fig. 2A, black circles). The effect of stimulus *l/|v|* on the probability of glide occurrence was significant (repeated measures binary logistic regression, Wald χ^2^ = 796.40, df = 11, p<0.001). Through the kite-like range of *l/|v|* ratios (vertical lines in Figs 2A−D), peak DCMD spike rates were relatively high and glide occurrence relatively frequent. Both responses increased as *l/|v|* declined within the kite-like range, but appeared to level off at low *l/|v|* values outside of this range.

We examined the timing of gliding responses to looming discs in order to assess whether glides occurred before collision within the kite-like range of *l/|v|* values. Mean glide timing was >150 ms in advance of collision at *l/|v|* 80.0 ms, but as looming disc *l/|v|* declined (because approaches became faster), mean glide timing shifted closer to the projected time of collision, occurring after the projected time of collision at *l/|v|* 5.3 ms and below (Fig. 2B; repeated measures ANOVA F_4.3,47.5_ = 42.85, p<0.001; *l/|v|* 80.0 ms excluded due to missing cases). The proportion of glides performed in response to a looming stimulus that were before the moment of collision is plotted in Fig. 2C. This figure shows that in response to an *l/|v|* 16.0 ms disc, >70% of glides were initiated before collision, but in response to an *l/|v|* 4.0 ms disc, <30% of glides were initiated before collision. Below *l/|v|* 4.0 ms, outside of the estimated kite-like range of *l/|v|* values, the proportion of glides elicited before collision was negligible. Figure 2D shows the probability that a stimulus with a particular *l/|v|* value will trigger a glide before collision – it combines a locust’s probability of performing a glide (which is highest for lower *l/|v|* values; Fig. 2A, black circles) with the probability that a glide will be initiated before collision (which is highest for higher *l/|v|* values; Fig. 2C). The proportion of stimuli to which a locust responded with a glide before collision was >40% for *l/|v|* 16.0 ms, and it declined to <20% for *l/|v|* 4.0 ms. Thus, glides before collision were most frequently observed in response to stimulus approaches at *l/|v|* values higher than those predicted for kite attacks, and almost never observed for approaches with *l/|v|* values lower than those predicted for kites. Within the estimated kite-like range, the probability of a glide before collision in response to a loom declined with declining *l/|v|* (indicative of increasing approach speed).

### Locust dcmd and Behavioural Responses to Looming Discs with Wings

**Figure 3:**
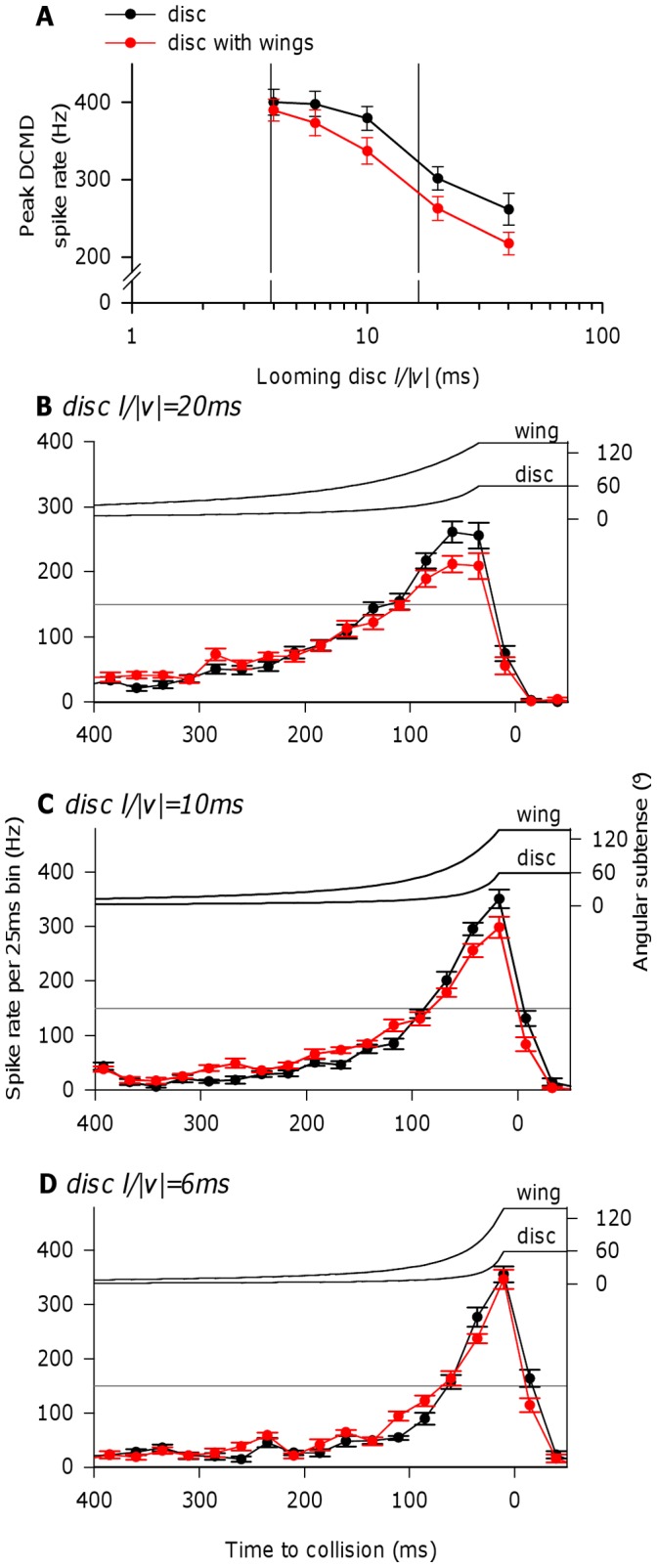
The effect on DCMD responses of adding wings to looming discs with varying *l/|v|*. A, Peak DCMD spike rate had a monotonic relationship with stimulus *l/|v|* for both looming discs, and looming discs with wings. However, the addition of wings to a looming disc caused a small but significant reduction in peak DCMD spike rate (see text). Vertical lines indicate range of kite-like *l/|v|* values, as in Figure 2. B–D, Mean DCMD spike rate plotted in 25 ms time bins for looming discs at three different *l/|v|* values, presented with or without additional wings. Calculated angular subtenses for the looming disc and wing tip are plotted above mean DCMD responses in each case. For disc *l/|v|* 20.0 ms (B), 10.0 ms (C) and 6.0 ms (D), the addition of wings had subtle effects on the DCMD response time course, slightly augmenting spike rates in the early stages of approach but decreasing them during the final, highest frequency part of the DCMD response (see text); these effects were most apparent at higher *l/|v|* ratios. Horizontal grey lines in each plot indicate the approximately 150 Hz threshold above which DCMD spikes can summate in order to trigger a glide [38]. This spike rate was achieved in response to looming discs with and without wings, but higher spike rates above the threshold were achieved earlier in response to looming discs without wings than looming discs with wings. Panels A–D: N = 10 locusts (value for each individual a mean of responses to 3 presentations of each stimulus). In B–D, symbols are aligned with the start of each 25 ms time bin. In all panels, means plotted ± SEM.

We next investigated the effect on DCMD responses and glide performance of adding wing-like extensions (‘wings’) to looming discs to produce a more bird-like stimulus shape. Wing length was constrained by the dimensions of our display screen to a span of 360 mm, or 4.5 × body width (wing span is approximately 10 × body width in a real kite). Adding wings to a looming disc depressed the peak DCMD spike rate for most disc *l/|v|* ratios (Fig. 3A; 2-way repeated measures ANOVA: effect of wings – F_1,9_ = 12.07, p = 0.007; effect of *l/|v|* – F_2.2,19.4_ = 117.39, p<0.001; interaction – F_2.4,21.4_ = 2.57, p = 0.092). At *l/|v|* 4.0 ms, the depression was relatively small (decrease in mean peak spike rate from 400 Hz in response to a looming disc, to 390 Hz in response to a disc with wings), but the depression was greater at higher *l/|v|* ratios (at *l/|v|* 40.0 ms, mean peak spike rate was 261 Hz in response to a looming disc, and 217 Hz in response to a disc with wings). Inspection of the DCMD response time courses for *l/|v|* 20.0, 10.0, and 6.0 ms looms shows that adding wings to a looming disc slightly augmented DCMD spike rates during the early stages of approach, but depressed them during the final, highest frequency part of the DCMD response close to the end of stimulus movement (Fig. 3B−D). However, the effect of wings in depressing DCMD spike rates was greater at low *l/|v|* (slower approach speeds). Over the final 250 ms of looming stimulus approach there was a significant interaction between effects of wings and time bin at all three *l/|v|* values examined, but a significant effect of adding wings alone only at *l/|v|* 20.0 ms (Fig. 3B–D; 2-way repeated measures ANOVAs: (i) *l/|v|* 20.0 ms: effect of wings – F_1,9_ = 11.99, p = 0.007; effect of time bin – F_2.1,19.2_ = 77.75, p<0.001; interaction – F_5.0,45.3_ = 5.25, p = 0.001. (ii) *l/|v|* 10.0 ms: effect of wings - F_1,9_ = 0.19, p = 0.670; effect of time bin - F_2.7,24.1_ = 230.33, p<0.001; interaction - F_3.7,33.2_ = 5.67, p = 0.002. (iii) *l/|v|* 6.0 ms: effect of wings - F_1,9_ = 2.77, p = 0.130; effect of time bin - F_3.5,31.9_ = 232.94, p<0.001; interaction - F_4.4,39.2_ = 3.66, p = 0.011).

**Figure 4:**
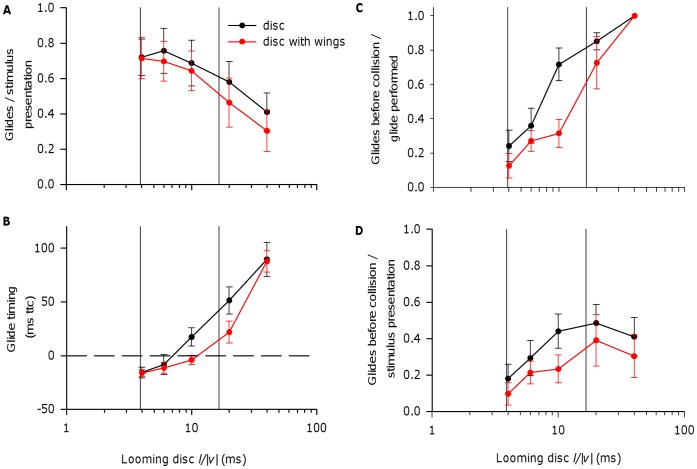
The effect on gliding responses of adding wings to looming discs with varying *l/|v|*. A, Adding wings to looming discs caused a small, non-significant decrease in the relative frequency of glide occurrence across *l/|v|* values (see text). In this and following panels, vertical lines indicate the estimated range of kite-like *l/|v|* values as in Fig. 2. B, The addition of wings to a looming disc appeared to delay the timing of glide initiation at *l/|v|* 10.0 and 20.0 ms (but not at 4.0 and 6.0 ms). Overall, addition of wings to a looming disc had a significant effect on glide timing. However, a significant difference (p<0.05) in glide timings between the winged and unwinged discs at a given *l/|v|* value was only found at *l/|v|* 10.0 ms (see text). Dashed line is the calculated time of collision. C, D, The addition of wings to a looming disc caused a small reduction in the proportion of glides that were successfully initiated before collision (C), and in the number of glides initiated before collision as a proportion of stimulus presentations (D). See text for statistical tests. Panel A, D: N = 10 locusts (value for each individual calculated from 5–6 presentations of each stimulus). Panels B, C: Varying numbers of individuals make up the data points at each *l/|v|* since locusts that did not glide could not be included. N at *l/|v|* 40.0 ms no wing = 7; wing = 5; *l/|v|* 20.0 ms no wing = 9; wing = 7; remaining *l/|v|*s with and without wings = 9. In all panels, means plotted ± SEM.

Gliding behaviour occurrence and timing is much more variable across repeated trials and individual locusts than the DCMD response is. Although locusts had a slightly lower probability of gliding in response to looming discs with wings than looming discs without wings at most disc *l/|v|* ratios (Fig. 4A), the effect was not significant (repeated measures binary logistic regression: effect of wings – Wald χ^2^ = 0.83, df = 1, p = 0.363; effect of *l/|v|* – Wald χ^2^ = 19.01, df = 4, p = 0.001; interaction – Wald χ^2^ = 2.63, df = 4, p = 0.622). Mean glide timing appeared to be later for looming discs with wings at *l/|v|* 10.0 and 20.0 ms, but not at *l/|v|* 4.0 and 6.0 ms (Fig. 4B). Overall, the effect of presence or absence of wings on glide timing was significant (2-way repeated measures ANOVA: effect of wings – F_1,6_ = 7.18, p = 0.037; effect of *l/|v|* – F_1.2,7.0_ = 21.27, p = 0.002; interaction – F_1.4,8.2_ = 1.78, p = 0.225; *l/|v|* 40.0 ms excluded due to missing cases). Using paired t-tests to detect significant differences in mean glide timing between the winged and unwinged disc at each *l/|v|* value, we found significant differences of p<0.05 only for *l/|v|* 10.0 ms, and no significant differences at any *l/|v|* value using Bonferroni adjusted p<0.0125 (Paired t-tests: *l/|v|* 20.0 ms – t = 1.95, df = 6, p = 0.100; *l/|v|* 10.0 ms – t = 2.86, df = 8, p = 0.021; *l/|v|* 6.0 ms – t = 0.31, df = 8, p = 0.766; *l/|v|* 4.0 ms – t = 0.12, df = 8, p = 0.910). Nevertheless, and as predicted by the overall trend in glide timing, adding wings to looming discs resulted in a slightly lower proportion of glides occurring before collision (Fig. 4C; Repeated measures binary logistic regression: effect of wings – Wald χ^2^ = 4.54, df = 1, p = 0.033; effect of *l/|v|* – Wald χ^2^ = 54.90, df = 3, p<0.001; interaction – Wald χ^2^ = 2.83, df = 3, p = 0.418; test excluded *l/|v|* 40.0 ms due to missing cases). As a result, adding wings to a looming disc also resulted in a lower proportion of stimulus presentations triggering glides that were initiated before collision (Fig. 4D; Repeated measures binary logistic regression: effect of wings – Wald χ^2^ = 4.29, df = 1, p = 0.038; effect of *l/|v|* – Wald χ^2^ = 62.38, df = 4, p<0.001; interaction – Wald χ^2^ = 3.66, df = 4, p = 0.454). Thus, adding wings to a looming disc appeared to have little effect on glide occurrence, but did have subtle effects on the timing of glide initiation.

## Discussion

Attacking birds are a natural predatory threat faced by locusts flying in swarms. Here we measured the ground speeds at which black kites attack flying locusts and estimated from these that the looming thoraces of kites, as viewed by locusts, are likely to be characterised by *l/|v|* ratios in the region of 4–17 ms. We investigated the previously identified relationships of DCMD peak spike rate [29], [30] and gliding response occurrence [37] with stimulus *l/|v|*, over an extended range of *l/|v|* values, allowing us to relate these trends to the characteristics of natural predator attacks. For both DCMD and gliding, strong responses occur in the *l/|v|* range estimated for attacking kites. The same overall pattern was observed when we added wings to simulated looming discs. However, adding wings to a looming disc caused small but significant effects on the DCMD response, in particular causing a slight reduction in spike rate during the final stages of stimulus approach. Addition of wings to looming discs also caused slight delays in glide initiation. Regardless of presence or absence of wings, glides were triggered closer to the time of collision as *l/|v|* declined, and occurred relatively infrequently before collision at the lowest *l/|v|* ratios tested. Thus, although glides are triggered reliably at *l/|v|* ratios estimated to characterise bird predator attacks, they have a relatively low probability of being successfully initiated before interception by the attacking predator. However, the performance of the DCMD in triggering gliding responses in this scenario is in line with expectations for last-ditch escape responses where the probability of achieving a successful escape is often relatively low, as we shall discuss.

Many bird species prey opportunistically on locusts and some, including black kites, capture flying locusts [44], [46], [47]. It was important to measure the speeds of these predators during real attacks because performance can vary considerably across contexts [54]. Nevertheless, the attack speeds we report for black kites are in general agreement with radar measurements of free-flying, migrating *M. migrans* (mean speeds of 11.9 m/s –13.8 m/s, depending on type of flight [55], [56]), and with calculated gliding performance for this species (9.0 m/s [57]). Kites generally attacked using slightly downward or flat trajectories, but it would be possible for them to converge upon a flying locust at a variety of angles of azimuth. We used the flight speeds we measured to estimate a range of possible closing speeds accounting for different angles of convergence, and this corresponded to an *l/|v|* range for the thoraces of attacking kites of 4 to 17 ms. As a bird attacks a locust, we would expect the locust to attempt to escape by steering before resorting to a glide as a last-ditch escape tactic [39], [40]. However, providing that locusts don’t fly substantially faster than they have been measured to while swarming [53], attacks should still fall within the *l/|v|* range we predict. This estimated range is at the lower end of *l/|v|* values used to challenge the DCMD neuron in many laboratory experiments (e.g. [29], [30], [31]). However, similar ranges of *l/|v|* values would be characteristic of aerial attacks by birds on insects in general. Although further data aren’t available for bird predators hunting flying locusts, barn swallows (*Hirundo rustica)* coarse on the wing for flying insects at 8.6 m/s when flying low and straight, and 6.8 m/s when flying higher and more erratically [58]; foraging common nighthawks (*Chordeiles minor*) fly at 6.5 m/s when hunting flying insects [59]. These flight speeds translate to *l/|v|* values of 1.3–7.5 ms for the barn swallow, and 2.9–16.8 ms for the common nighthawk (using thorax widths of 0.033 m for *H. rustica* and 0.064 m for *C. minor*, calculated as 10% wingspan [60], [61]; prey flight speed of 4.6 m/s (equivalent to *L. migratoria*); and not accounting for standard deviation in either case).

A kite might converge upon a flying locust from any angle around the body in the horizontal plane, but in laboratory experiments all stimuli were delivered at 90° to the locust’s long axis due to constraints imposed by our experimental set up (which included a large fan apparatus for creating airflow). The angle of approach for a looming stimulus does not affect the timing of peak DCMD response, except at the extreme periphery of the receptive field [62], [63], [64]. Peak spike rate is largely equivalent for angles of approach from 30–150° azimuth and from −15–+45° elevation, but decreases markedly outside of this region [64]. Thus, DCMD response would be largely equivalent for looms along most angles of azimuth. However, our approach will overestimate DCMD responses to the lowest *l/|v|* (head-on) and highest *l/|v|* (approaches from behind) looms. Relative to the solitarious phase, gregarious *S. gregaria* have a region of pronounced DCMD sensitivity caudal and slightly dorsal of the eye’s centre [64]. Video footage showed kites circling above swarming locusts, swooping down into the swarm to make an attack, and the DCMD receptive field may be well suited to detecting such an attack strategy. Steering responses during bird attacks might cause the apparent motion of an attacking kite to consist of periods of translation as well as looming. However, when objects undergo a period of translation before looming, there is no effect on looming-elicited peak DCMD spike rate, and only a small effect on peak timing for some stimulus configurations [63].

Our results in *L. migratoria* support the previously published relationship between peak DCMD spike rate and looming stimulus *l/|v|* demonstrated for *Schistocerca americana*, and in both solitarious and gregarious *S. gregaria* (Acrididae: Cyrtacanthacridinae) [29], [30]. The monotonic increase in peak DCMD spike rate with declining stimulus *l/|v|* is reflected in the frequency of glide occurrence, which follows a broadly similar pattern (as we reported previously in a different set of experiments [37]). Superimposing our estimated bird predator-like range of *l/|v|* values onto these relationships, it is apparent that both DCMD and behavioural responses are strong to stimuli within this naturalistic range. However, it would be premature to discuss how such a stimulus-response relationship may have evolved without first appreciating the importance of bird predators as a selective force (e.g. [4]). Birds are a natural predator of locusts (and acridids more generally), and the video recordings we analysed support the assertion that they can take considerable numbers of locusts when they are aggregated together in a swarm. However, the impact of such predation on locust populations is highly variable [44], [46], and for an individual locust, membership of a swarm can confer a degree of protection from predator attack [65]. Furthermore, the same relationship between DCMD response and *l/|v|* exists for solitarious and gregarious *S. gregaria* [30] which differ in flight behaviour as well as population density and, therefore, possible vulnerability to aerial predators. Finally, the DCMD is a multi-functional neuron implicated in emergency behavioural responses on the ground and in flight [32], [33]. A range of different predators may be experienced across these very different circumstances. Taking all these factors into account, it is certainly possible that flying bird predators are a selective pressure on the response properties of the DCMD, but it seems unlikely that they are the only one.

Nevertheless, birds are a verified natural predator of swarming, flying locusts so the performance of the DCMD in eliciting glides to bird-like looms remains a valid area for investigation. Glides were triggered closer to collision at lower *l/|v|*, similar to locust jump preparation and triggering [34], [36], and escape and defensive responses to simple looming stimuli in other species [66], [67]. Within the range of *l/|v|* values predicted for bird predators, there was an approximately 50–80% probability that a locust would trigger a gliding response to a looming stimulus, and an approximately 15–80% probability that when a glide was triggered it would be successfully initiated before collision. Studies of fast escape systems in other taxa show broadly comparable performance. For an adult wood cricket challenged from the side with a piston producing air movements that mimic a wolf spider attack, there is an approximately 60% probability that an escape response will be elicited, and a 50% probability that that escape will be successful [6]. However, both probabilities vary considerably with the angle from which an attack comes, and escape responses are elicited more frequently in juvenile crickets which experience greater predation by wolf spiders [4], [6]. When juvenile crayfish perform lateral and medial-giant triggered tail flips to evade an attacking dragonfly nymph, they successfully evade the firm grasp of the dragonfly nymph in 45–50% of cases (but in only around 20% of cases for non-giant tail flips) [7]. Furthermore, it is crucial to recognise that locusts steer away from a looming threat as their primary means of evading it [37], [39], [40], [68]. As such, glides are resorted to only as a last-ditch tactic when steering has not been successful, and we would not expect such behaviours to result in a high degree of success. During free-flight encounters with bats, mantids had a 76% escape probability, but when deafened and reliant on last-ditch tactics (including those triggered by air movements detected by their cercal system), escape probability declined to 34% [69].

In general agreement with a locust’s probability of gliding before collision from laboratory experiments, our observations from video revealed that black kites consumed a locust after about 80% of attacks, indicating a high probability of capture success. In contrast, dragonfly nymphs were only successful in killing juvenile crayfish in <20% of attacks, despite the relatively low success of the crayfishes’ initial evasive response [7]. This is because subsequent tail flips following capture could free a crayfish from a dragonfly nymph’s grasp [7]. For a locust, there may be a very low probability of escape once firmly grasped in the talons of a kite, and a combination of steering and gliding may provide the locust’s best chance of successful escape.

The functional consequences of a glide are difficult to infer. A related grasshopper in the family Oedipodinae, *Dissosteira carolina*, has been observed making sudden dives to the ground from flight to evade chasing birds [70], and this is one possible interpretation of the gliding response. However, in response to looming stimuli in the estimated bird-like range of *l/|v|* values, glides occur only a short time before collision and might not be capable of achieving more than a small degree of course change before collision (see also [37]). During their free-flight encounters with bats, deafened mantids with operational cerci were dropped more often than deafened mantids without operational cerci [69], indicating that the main benefit of last-ditch defence triggered by the cercal system may be in increasing the likelihood of mis-handling by the predator through a relatively small, late deviation in course. This seems the most likely benefit conferred by a last-ditch glide in response to an attacking bird, and our video recordings provided anecdotal evidence that kites do mishandle and drop locusts during attempted capture, as well as completely missing the target locust at times. Although it has been suggested that glides may be preparation for an evasive banked turn in *S. gregaria* [41], glides are too long (mean duration >130 ms for glides after which flight resumes [37]), and occur too close to collision, to fulfil such a function.

Adding wing-like extensions to a looming disc had little effect on the overall relationships with stimulus *l/|v|* of DCMD response and glide performance discussed so far. Although ours is not the first study to use bird-like shapes to stimulate the DCMD, the only previous study focussed on habituation of the DCMD response and did not experimentally compare simple versus complex looming shapes [52]. Since the performance of visual pathways in response to naturalistic stimuli can differ from that predicted from responses to simple stimuli [71], [72], it is reasonable to ask whether the DCMD and gliding performance are adequately stimulated by simple looming discs. However, the addition of wings to a looming disc actually induced slightly weaker DCMD responses, and slightly delayed glide initiation. A reduction in DCMD peak spike rate may be due to an increase in the effects of lateral inhibition between elements presynaptic to the LGMD [25], [28], [73], caused by a relative increase in the extent of the moving edges in the image, and the expansion of the wing tip in advance of the expansion of the main disc. Summation of DCMD spikes above a threshold of 150 Hz is implicated in glide triggering [38], and this threshold was reached in response to looming discs with and without wings. However, the steeper rise in spike rate above this threshold in response to a disc without wings means that summation sufficient for a motor neuron spike would be achieved earlier, leading to earlier glide initiation. Trends in DCMD response profile and glide timing were most evident at higher *l/|v|* values within the tested range, towards the upper end of that estimated for kite attacks. However, gliding occurrence and timing are relatively variable due to interactions between DCMD response and the ongoing wingbeat cycle [38], and small delays in glide timing are indicated across *l/|v|* values by a decrease in the probability of glide occurrence before collision in response to discs with wings versus discs without wings. It is important to note that the wing we used was shorter, relative to disc diameter, than the wing of a real kite and did not flap. This was a reasonable initial approach because kites often attacked with wings held still, outstretched, and slightly flexed at the wrist during a gliding dive.

In this paper we have provided evidence that the attacks of an avian predator of flying locusts are likely to be characterised by relatively low *l/|v|* ratios for the looming bird’s thorax. In response to looming discs with similar *l/|v|* ratios, the DCMD responds strongly, and glides occur readily with probabilities of glide initiation before collision equivalent to successful escape rates in other last-ditch escape systems. Adding outstretched wings to looming discs for a more bird-like profile has subtle effects on DCMD response and gliding performance. Further studies of predator-prey interactions in a natural context are essential to advance our understanding of this and other escape-triggering neural mechanisms.

## Materials and Methods

### Ethics Statement

Experiments conformed to the legal requirements of the UK and Republic of Ireland which do not regulate experimentation on insects. Measurements of the black kite were obtained with the assistance, and under the guidance, of a trained falconer, and with permission from the International Centre for Birds of Prey (Gloucestershire, UK).

### Black Kite Behaviour

Video of black kites (*M. migrans)* capturing flying Australian plague locusts, *C. terminifera* (Acrididae: Oedipodinae), was obtained from Natural History New Zealand (Dunedin, New Zealand). Footage was recorded in Mundi Mundi, NSW, Australia, in the summer of 2000. Frame resolution was 575×720 pixels.

In some footage, kites were filmed from a distance with a static camera (e.g. Fig. 1A). This was only used to qualitatively describe the overall pattern of kite attack behaviour. Large numbers of kites (20–25 in frame at times) circled above swarming, flying locusts, periodically swooping into the swarm to make a catch.

In other footage, the camera panned to follow single kites through multiple swooping attacks (e.g. Fig. S1). This was viewed using VirtualDubMod (http://virtualdubmod.sourceforge.net/) and inter-attack intervals measured for 12 individual kites that were continually in frame for 17–109 s each and made ≥ three attacks each. Capture success was measured as the frequency at which attacks were followed by the kite transferring a prey item from talons to beak (excluding cases in which this behaviour may have been obscured from view). For analysis of kite ground speeds, sequences of frames were extracted, de-interleaved using ImageJ (http://imagej.nih.gov/ij/index.html), and combined as an image stack using Image Tool (UTHSCA, San Antonio, TX, USA; http://compdent.uthscsa.edu/dig/itdesc.html). We chose 17 attacks by 10 individuals in which the camera panned to follow a kite through an attack oriented perpendicular to the camera (see Fig. 1B). The x, y co-ordinates (pixels) of the kites’ beaks were marked relative to clearly identifiable image features where they were available (because the camera panned to follow the attack; e.g. Fig. S1). This meant that several segments may have been analysable within each attack sequence. Measurement error was reduced by making each measurement three times and taking the mean. The length of an individual kite was 69.5±15.0 pixels across sequences and was used to calibrate the co-ordinate system to published size measurements (wingspan = 1.20–1.53 m, length = 0.46–0.66 m [51]; means used for calibration and calculation: wingspan = 1.36 m, length = 0.56 m). In order to avoid pseudoreplication, mean speeds for each analysable segment were calculated as the mean of speed estimates at each frame within that segment. Mean speed for each attack was the mean across all segments in that attack, and mean speed for each kite the mean across all attacks by that kite.

We also filmed a single, trained, male black kite at the International Centre for Birds of Prey (Newent, Gloucestershire, UK), where we could make recordings with a static video camera and directly measure features in frame for more exact calibration than was possible in video of wild kite attacks. The trained kite was filmed catching food items thrown into the air by a falconer, but on later analysis of videos this behaviour was noted to be qualitatively different to that seen in wild kites. Nevertheless, videos of the trained kite provided useful support for the speed estimates made for wild kites, and helped in the assessment of deceleration trends during wild kite attacks.

In order to characterise black kite attacks from the locust point of view we needed to convert kite attack speeds to closing speeds with the target locust. However, the target locust could not be seen in most attack sequences. Using published flight speed measurements for *L. migratoria* [53], we calculated closing speeds for a fast kite (mean speed + SD) and a fast locust (mean speed + SD) converging head-on, and for a slow kite (mean speed – SD) converging on a fast locust (mean speed + SD) from behind. The geometry of a looming disc is described by its *l/|v|* ratio, so we calculated this ratio for a looming kite’s thorax, which is approximately disc shaped when viewed head-on. Images suggested that kite thorax width was ∼10% of wingspan (0.136 m, see above), so we used *l* = 0.068 m for our calculations. We confirmed that this estimate of thorax width was reasonable by measuring a captive male black kite at the International Centre for Birds of Prey. This bird had a wingspan of 1.42 m, and a thorax width of 0.11 m.

### Locust dcmd and Behavioural Responses

Experiments were performed on adult *Locusta migratoria* L. (Acrididae: Oedipodinae), obtained from Blades Biological (Edenbridge, Kent, UK).

### (I) Visual Stimulation

Looming visual stimuli were programmed using Visionegg software [74] (http://www.visionegg.org/) on an Intel Pentium 4-equipped PC with a PNY (Parsippany, NJ, USA) Nvidia Geforce 6200 AGP8X graphics card. Stimuli were displayed on an Iiyama (Tokyo, Japan) visionmaster pro 454 HM903DT A CRT monitor running at a resolution of 640 × 480 pixels at 200 Hz.

Stimuli simulated the approaches of a 0.08 m diameter black disc at 0.5–15.0 m/s. A 0.08 m diameter looming disc can be used to represent a 0.136 m diameter kite body looming at a faster speed because the expansion profile of looming objects with the same *l/|v|* ratio is identical. By altering disc approach speed we produced disc *l/|v|* values of 80.0, 40.0, 26.7, 20.0, 16.0, 13.3, 10.0, 8.0, 6.0, 5.3, 4.0, 3.2, and 2.7 ms, allowing us to sample DCMD and behavioural responses within and to both sides of the kite-like *l/|v|* range we estimated. Looming disc stimuli were delivered with or without a 0.36×0.02 m horizontal black bar representing wings intersecting the disc 0.018 m above its centre. Stimuli were presented over a white background. Background luminance was estimated at 65.7 cd/m^2^ (using Canon (Tokyo, Japan) IXUS 850IS digital camera spot meter, after Unwin [75]). This is broadly comparable to the luminance of a grey sky near to the horizon at noon (100 cd/m^2^ [76]), and is similar to that used in some previous studies of the locust DCMD (e.g. [77]). Objects approached over a simulated distance of 10 m and ended their approach level with the monitor screen, 0.07 m from the locust. A custom-built light-detector circuit monitored an area of screen that dimmed during stimulus delivery for data synchronisation.

### (ii) DCMD Recordings

DCMD activity was recorded extracellularly in 20 locusts. Locusts were restrained ventral side up using plasticine bands. The head was restrained using plasticine and insect pins and the ventral sclerite of the neck exposed by tilting the mouthparts forward. A pair of 50 µm copper wires, insulated but for their tips, were inserted through a pair of holes pierced on the left-hand side (relative to the locust) of the neck sclerite. DCMD recordings were amplified with standard AC amplifier and captured to disc using a micro 1401 analogue-to-digital converter and Spike2 v. 6 for Windows (Cambridge Electronic Design, UK). In our first experiment, each locust received six presentations of looming discs at twelve *l/|v|* values. In our second experiment, each locust received three presentations of looming discs with and without wings at five *l/|v|* values. In both experiments stimuli were delivered in pseudorandom order and separated from the next by a 2.5 min interval. During the interval, a hindleg was mechanically stimulated for 5 s to prevent habituation and to ensure a flight-like arousal of DCMD sensitivity [31]. DCMD spikes were the largest in the nerve cord and were identified offline using Spike2. DCMD responses were converted to a mean spike rate computed at each spike event and calculated by averaging over the preceding 25 ms (half wingbeat) window. An individual’s peak DCMD spike rate was calculated as the mean across all stimulus presentations at each *l/|v|*.

### (iii) Flight Behaviour

Glide occurrence was recorded in 25 flying locusts tethered via the dorsal pronotum to a metal bar suspended in front of a laminar airflow (see [37]). An infra-red emitter and detector circuit produced a voltage signal each time a wing passed a horizontal line parallel with the locust’s long axis. Locusts were allowed to fly until they adopted strong flight and a characteristic flight posture (normally with hind legs tucked). Stimuli were as described above for electrophysiological recordings. Each locust received five or six presentations of each stimulus delivered in pseudorandom order and separated by an interval of 30 s (the DCMD is robust to habituation in flying locusts, even at lesser inter-stimulus intervals [31]). If a locust did not fly strongly during a stimulus presentation, that presentation was excluded from further analysis. Glides were defined as pauses between consecutive wingbeats >1.25×mean duration of the preceding 10 wingbeats [37]. Glide timings were calculated using the timing of the last signal from the IR wingbeat sensor preceding each glide, relative to the signal from the light-detector circuit monitoring stimulus delivery.

### Statistical Analysis

Statistical analyses were conducted using IBM SPSS Statistics v. 19.0 (IBM Corp., Armonk, NY, USA). Electrophysiological and glide timing data were analysed using one- (*l/|v|*) or two-factor (*l/|v|* and presence or absence of wings) repeated measures ANOVA. Sphericity was assessed using Maunchly’s test and evaluation of epsilon. Where violations were detected by either metric, the Greenhouse-Geisser correction to degrees of freedom was applied. Glide occurrence data were analysed using repeated measures binary logistic regression implemented using the SPSS ‘GENLIN’ procedure. We report test of model effects statistics describing whether a tested factor is a significant predictor of glide occurrence. Because glides did not occur with the same frequency in response to each stimulus, our data set for measurements of those glides (timing, probability of triggering before collision) was unbalanced. To conduct statistical analyses of these measurements, we therefore excluded stimuli to which glides were rare; listwise deletion within SPSS then removed individual locusts with missing cases from the analysis.

## Supporting Information

Figure S1 **A typical attack by a black kite (** ***M. migrans*** **), on a flying locust (** ***C. terminifera*** **).** Images proceed left to right, and top to bottom, and in each frame the original recording timecode is provided (hours:minutes:seconds:frames; 25fps). Here, a locust is captured immediately after the last frame in the sequence. Since the camera panned to follow kites through each attack, distinctive image features were used to centre a coordinate system for speed measurements (in this case, a tree; white dot with black edge). © NHNZ Moving Images.
(TIF)

Figure S2 **Sudden height loss by a flying locust in response to a looming black kite.** In this sequence a black kite swoops (frames A–D), and intercepts a locust (frames E and F, target locust not visible), but a second locust is clearly visible in the same focal plane (arrows). The kite (not now attacking), looms behind the steadily flying locust (frames G–J). The locust then quickly looses height when the kite gets close (frames K–P). Images proceed left to right, and top to bottom and are enlargements of the same section of each frame; inter-frame interval is 40 ms. Locusts are Australian plague locusts (*C. terminifera*). © NHNZ Moving Images.
(TIF)
