# Supplementary figures and images for: Predator versus Prey: Locust Looming-Detector Neuron and Behavioural Responses to Stimuli Representing Attacking Bird Predators
Source: PLoS One. 2012 Nov 27;7(11):e50146. doi: 10.1371/journal.pone.0050146 (PMC3507823; doi:10.1371/journal.pone.0050146)

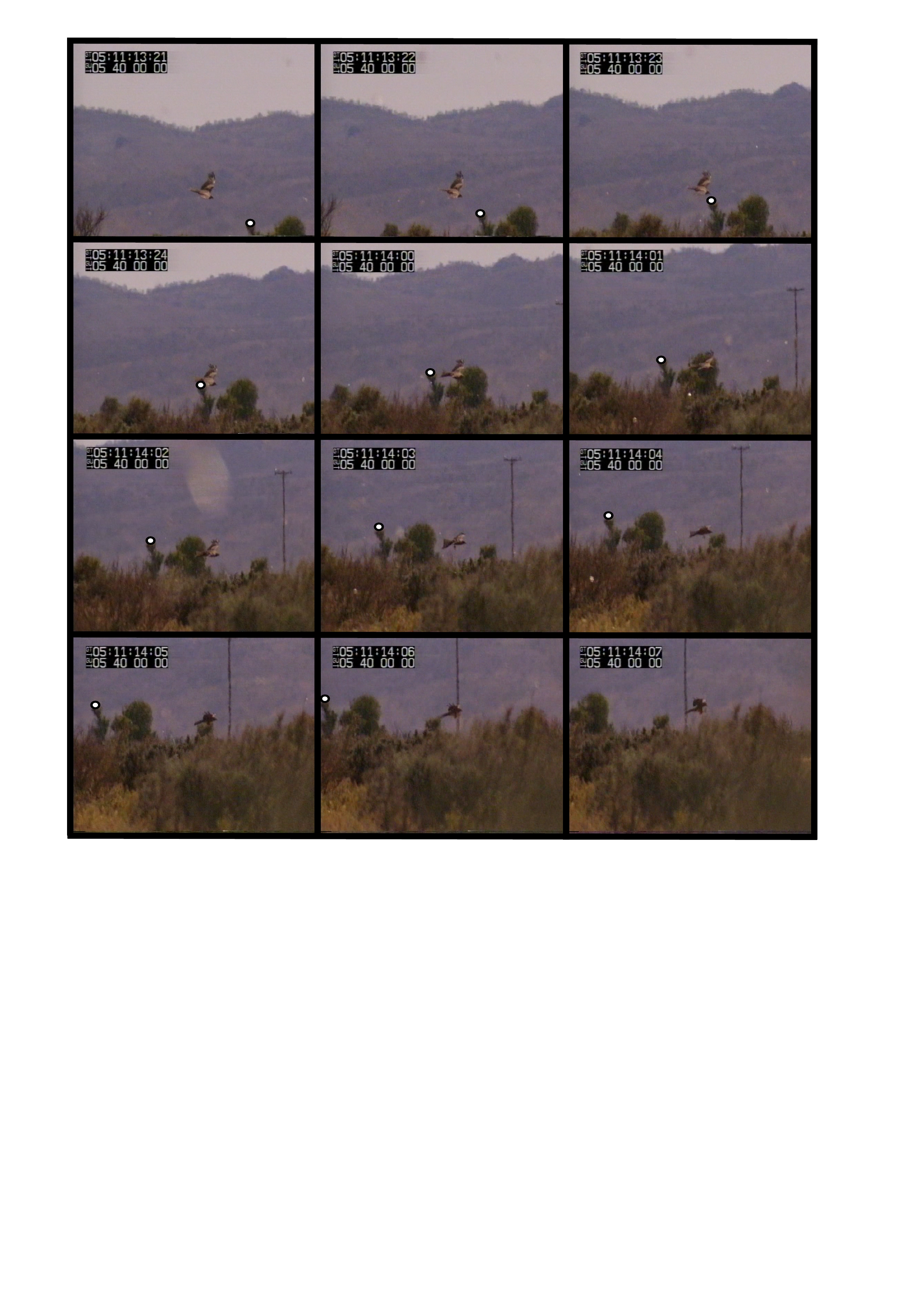

Supplement: Figure S1 — A typical attack by a black kite ( M. migrans ), on a flying locust ( C. terminifera ). Images proceed left to right, and top to bottom, and in each frame the original recording timecode is provided (hours:minutes:seconds:frames; 25fps). Here, a locust is captured immediately after the last frame in the sequence. Since the camera panned to follow kites through each attack, distinctive image features were used to centre a coordinate system for speed measurements (in this case, a tree; white dot with black edge). © NHNZ Moving Images. (TIF) [file pone.0050146.s001.tif]

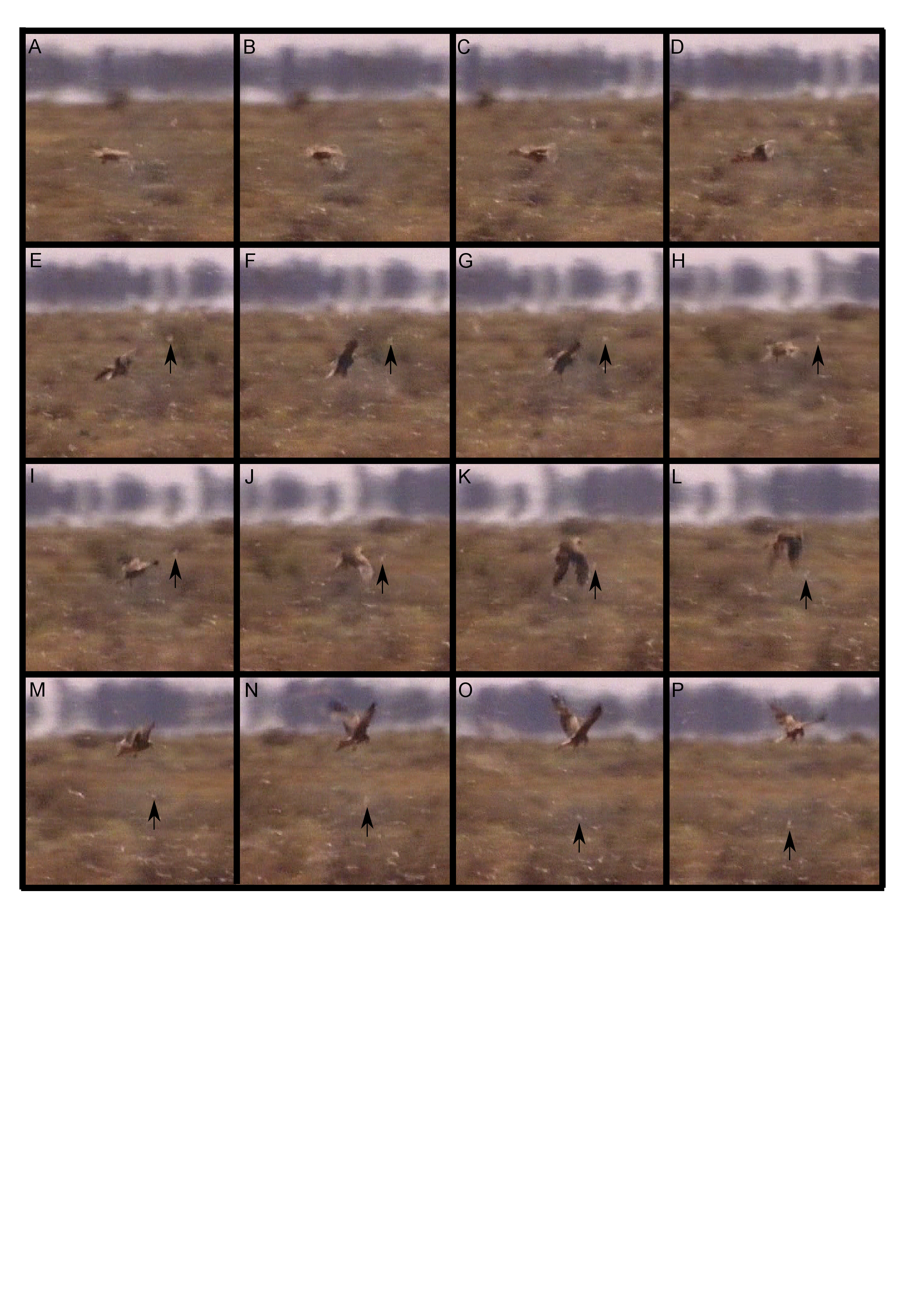

Supplement: Figure S2 — Sudden height loss by a flying locust in response to a looming black kite. In this sequence a black kite swoops (frames A–D), and intercepts a locust (frames E and F, target locust not visible), but a second locust is clearly visible in the same focal plane (arrows). The kite (not now attacking), looms behind the steadily flying locust (frames G–J). The locust then quickly looses height when the kite gets close (frames K–P). Images proceed left to right, and top to bottom and are enlargements of the same section of each frame; inter-frame interval is 40 ms. Locusts are Australian plague locusts (C. terminifera). © NHNZ Moving Images. (TIF) [file pone.0050146.s002.tif]
